# Complete genome sequence of a haloalkaliphilic heterotrophic bacterium *Halomonas salifodinae* IM328

**DOI:** 10.1128/mra.00084-24

**Published:** 2024-06-11

**Authors:** Zheyi Wang, Niping Yang, Mengshuang Liu, Dahe Zhao, Hua Xiang, Yanning Zheng

**Affiliations:** 1 State Key Laboratory of Microbial Resources, Institute of Microbiology, Chinese Academy of Sciences, Beijing, China; 2 University of Chinese Academy of Sciences, Beijing, China; 3 School of Life Sciences, Hebei University, Baoding, China; Indiana University, Bloomington, Bloomington, Indiana, USA

**Keywords:** genome, DNA sequencing, *Halomonas salifodinae*, compatible solutes

## Abstract

The genome of a halophilic bacterium *Halomonas salifodinae* IM328 was completely sequenced in order to offer convenience for the research such as the synthesis of compatible solutes. The genome contains a circular chromosome which was sequenced by PacBio system.

## ANNOUNCEMENT

*Halomonas salifodinae* IM328, which is a Gram-negative haloalkaliphilic heterotrophic bacterium, was previously isolated from the sediment of Yanhaizi Lake, Inner Mongolia, China; 108°26′14″N, 40°9′6′E (1). The sample was diluted and spread on HM-3.6 solid medium plates containing the following (g/L): sodium chloride 36, potassium chloride 2, magnesium sulfate heptahydrate 1, calcium chloride 0.27, sodium bromide 0.23, sodium bicarbonate 0.06, peptone 5, yeast extract 10, ferric chloride 0.001, pH 8.5 (adjusted with NaOH), and agar 1.5% (wt/vol) for solid medium. As a halophilic bacterium that could reach high optical density at 600 nm (above 2.0 with flask) and tolerate relatively high salinity, IM328 has the potential for a high yield of compatible solutes such as ectoine (2) and betaine (3). Since extremophiles have become a popular chassis for industrial bioproduction of high-value chemicals such as hydroxyectoine (4), which is costly to produce through chemical catalysis, complete genome sequencing is required for proper genetic modification in *H. salifodinae* IM328.

The whole genome sequencing was performed at Azenta Genewiz Biotech Co. Ltd, China with Illumina NovaSeq6000 platform and PacBio Sequel lle system. For genome DNA preparation, the cells were incubated at 37℃ overnight in 80 Luria-Bertani liquid medium, which contains 10.0 g/L of tryptone, 5.0 g/L of yeast extract, and 80.0 g/L of NaCl. Cells cultured in 10 mL liquid medium were collected. The genome DNA was extracted by HiPure Soil DNA Kit (Magen, China). For NovaSeq6000 platform, 100 ng gDNA was randomly broken by Covaris S220 and repaired by End Prep Enzyme Mix using VAHTS Universal Plus DNA Library Prep Kit for Illumina V2 (Vazyme, China). The concentration and quality of the library were examined by Qubit 3.0 Fluorometer (Invitrogen, Carlsbad, CA, USA) and Agilent 2100 Bioanalyzer System, successively. The read length of Illumina NovaSeq6000 platform was set to 2 × 150 bp, and fastp (v0.23.0) (5) is used to remove the adapter or primer sequences and reads <75 bp for quality control. The reads with Q20 <40% were also removed using fastp. Clean reads (40,047,768) (from 40,470,970 total reads) and 6,000,990,546 clean bases were obtained. For PacBio Sequel lle system (6), 5 µg gDNA was randomly broken using PacBio Covaris g-Tube DNA Shearing for SMRTbell Prep Kit 3.0, followed by quantification using Qubit 3.0 Fluorometer. The size of the library was checked to be appropriate using Agilent 2100 Bioanalyzer System before sequencing following the instructions of the manufacturer. A total of 21,341 sequences (average length: 7,603.45 bp) were obtained with an *N*50 value of 8,745.

PacBio reads were assembled using Hifiasm (v0.13-r308) (7) and Canu (v1.7) (8) with default settings for identifying the overlaps and trimming of mismatching bases, followed by recorrecting with software Pilon (v1.2.2) (9) using Illumina raw data. Out of a total of 21,341 reads, 21,339 reads were mapped to the genome with a coverage of 100% with a mean depth of 41× and assembly *N*50 value of 3,952,647. The genome assembly contains a single chromosome with a length of 3,952,647 nucleotides (66.5% guanine-cytosine content), and the origin of the circular genome was rotated to the start of chromosomal replication initiator protein gene *DnaA*. The sequence was deposited in the GenBank under the accession number CP141893.

## Data Availability

Sequences are available in NCBI databases under BioProject PRJNA1057535, Biosample SAMN39136389, and GenBank CP141893 (chromosome), and the Pacbio raw data and Illumina raw data have been deposited in SRA with the accession numbers SRR28162546 and SRR28904962.
